# Associations of Patient Mood, Modulators of Quality of Life, and Pharmaceuticals with Amyotrophic Lateral Sclerosis Survival Duration

**DOI:** 10.3390/bs10010033

**Published:** 2020-01-10

**Authors:** Leila Bond, Gloria Bowen, Benjamin Mertens, Keelie Denson, Kathleen Jordan, Branislav Vidakovic, Cassie S. Mitchell

**Affiliations:** 1 Department of Biomedical Engineering, Georgia Institute of Technology & Emory University, Atlanta, GA 30332, USA; lbond9@gatech.edu (L.B.); gbowen6@gatech.edu (G.B.); b3njam1nm3rt3n5@gmail.com (B.M.); kdenson@augusta.edu (K.D.); kjordan37@gatech.edu (K.J.); 2 School of Medicine, Medical College of Georgia, Augusta University, Augusta, GA 30912, USA; 3 Department of Industrial & Systems Engineering, Georgia Institute of Technology, Atlanta, GA 30332, USA; brani@gatech.edu

**Keywords:** ALS, quality of life, survival, mood, antidepressants, off-label drugs, supplements

## Abstract

Associations of modulators of quality of life (QoL) and survival duration are assessed in the fatal motor neuron disease, Amyotrophic Lateral Sclerosis. Major categories include clinical impression of mood (CIM); physical health; patient social support; and usage of interventions, pharmaceuticals, and supplements. Associations were assessed at *p* < 0.05 and *p* < 0.001 significance thresholds using applicable methods (Chi-square, *t*-test, ANOVA, logistical regression, random forests, Fisher’s exact test) within a retrospective cohort of 1585 patients. Factors significantly correlated with positive (happy or normal) mood included family support and usage of bi-level positive airway pressure (Bi-PAP) and/or cough assist. Decline in physical factors like presence of dysphagia, drooling, general pain, and decrease in ALSFRS-R total score or forced vital capacity (FVC) significantly correlated with negative (depressed or anxious) mood (*p* < 0.05). Use of antidepressants or pain medications had no association with ALS patient mood (*p* > 0.05), but were significantly associated with increased survival (*p* < 0.05). Positive patient mood, Bi-PAP, cough assist, percutaneous endoscopic gastrostomy (PEG), and accompaniment to clinic visits associated with increased survival duration (*p* < 0.001). Of the 47 most prevalent pharmaceutical and supplement categories, 17 associated with significant survival duration increases ranging +4.5 to +16.5 months. Tricyclic antidepressants, non-opioids, muscle relaxants, and vitamin E had the highest associative increases in survival duration (*p* < 0.05). Random forests, which examined complex interactions, identified the following pharmaceuticals and supplements as most predictive to survival duration: Vitamin A, multivitamin, PEG supplements, alternative herbs, antihistamines, muscle relaxants, stimulant laxatives, and antispastics. Statins, metformin, and thiazide diuretics had insignificant associations with decreased survival.

## 1. Introduction

Amyotrophic lateral sclerosis (ALS) is a progressive neurodegenerative disease characterized by the death of motor neurons. ALS results in difficulty swallowing, paralysis, and respiratory dysfunction [1]. Riluzole and edaravone are the two primary medications prescribed to slow the underlying ALS etiology and are the only two currently approved by the United States Food & Drug Administration. At best, both medications slow disease progression for only a few months [2].

Since ALS is a debilitating and fatal disease, quality of life (QoL), an individual’s sense of well-being and ability to carry out activities of daily living, is a major concern and driver of clinical care [3,4]. Due to the limited efficacy of ALS etiological drugs, palliative interventions are commonly used to alleviate non-curable ALS symptoms with the purpose of improving QoL and/or extending survival duration. Interventions largely consist of modes to manage symptoms such as gastrostomy tubes to provide nutritional supplementation in patients with dysphagia; noninvasive ventilation (NIV) such as bi-level positive airway pressure (Bi-PAP) to improve respiratory efficiency [5]; equipment to assist in ambulation such as leg braces, walkers, or wheelchairs; oral nutritional supplements like vitamins, canned meal supplements, or alterative herbs; and palliative pharmacologic treatments, such as antidepressants, antianxiety, antispastics, anticholinergics, and pain medications [6]. Theoretically, these therapeutic interventions aid patients in living a more comfortable and fulfilling life, which is hypothesized to improve patient mood. Studies have demonstrated that poorer mood is correlated with faster disease progression and shorter survival duration [7,8]. Thus, palliative therapeutic interventions are hypothesized to indirectly increase survival duration [7,9,10]. 

Not surprisingly, both functional metrics and QoL metrics are useful in assessing the overall impact of ALS, both physically and psychologically, in the context of patient care and clinical trials [11]. Several generic QoL instruments are utilized to assess patients with ALS, such as the McGill Quality of Life Scale (MQOL), Short Form 36 (SF-36), the Neurology Quality of Life (Neuro-QoL) Measurement System, the Sickness Impact Profile, the Schedule for the Evaluation of Individual Quality of Life (SEIQoL), among many others [11,12,13,14,15,16]. There are a few ALS disease-specific instruments used as well. The revised ALS Functional Rating Scale (ALSFRS-R) provides an overall measure of functional disease progression [17], which encompasses respiratory function and activities of daily living. In contrast, the ALS Assessment Questionnaire-40 (ALSAQ-40) and the ALS-Specific Quality of Life Questionnaire (ALSSQOL) [11,14,15,18] focus on quality of life.

The existence of numerous instruments reflects the complexity of QoL and how difficult it is to measure due to its subjective and multifactorial nature, as well as the diverse definitions for QoL [19]. QoL assessment is further complicated in ALS due to underemployment of QoL assessments, which are often lengthy, take much effort to compete, and can be skewed by patient or caretaker bias [20]. There is also a lack of standardized QoL instruments in the clinical setting and, some believe, overemphasis of strength and physical factors among current instruments [18,21,22,23,24,25]. Rather than using a survey, this study utilizes a newer metric, clinical impression of mood (CIM) [20]. CIM relies on clinician evaluation of patient mood during each visit using objective verbal and non-verbal cues, as well as subtle subjective cues based on the clinician’s consistent, long-term relationship with the individual patient over three or more visits. CIM uses text mining of the electronic medical record to map specific adjectives to categorize the mood as either “positive” or “negative” [20]. Past research lends support to the feasibility of such a metric, as depression, for example, has several facial and vocal biomarkers that can be easily assessed [26,27].

An important component in QoL is the patient’s mood throughout the course of the disease. There has been some evidence that higher QoL improves survival in patients with terminal prognoses [28]. There is much interest in the impact of mood-stabilizing drugs on patient QoL and survival. However, there is evidence that clinical depression among ALS patients is not as widespread as might otherwise be expected [29], and that antidepressants do not always significantly change ALS patients’ moods [30]. In fact, impact of antidepressant is controversial based on preclinical mouse model experimental results [31]. For example, some antidepressants have been shown to delay disease onset and extend the lifespan of mouse models with ALS and other neurodegenerative diseases [32,33]. Selective serotonin reuptake inhibitors (SSRIs) have specifically shown preclinical pro-cognitive effects [34]. However, antidepressant usage is not ubiquitously positive. The (SSRIs) sertraline and paroxetine reduced astrocyte viability, induced dose-dependent intracellular calcium elevation, and activated apoptosis in primary astrocyte and neuron co-cultures [35]. In a clinical setting, antidepressant users were more likely to develop dementia than non-users, regardless of their depressive state [36]. There is not enough conclusive scientific evidence to determine whether the potential QoL or neuroprotective effects of antidepressants are strong enough to improve survival in ALS patients.

Beyond antidepressants, other prescription or over the counter palliative drugs are used to treat some symptoms of ALS, such as excessive secretions or drooling (e.g., with anticholinergics), muscle spasticity or fasciculation (e.g., with antispastics like baclofen), supplements for nutritional support or to combat ALS-related oxidative stress (e.g., multivitamins or specific vitamins like vitamin E), and pain medications (e.g., ibuprofen, acetaminophen, and limited use of some opioids). ALS patients generally have less antecedent conditions (defined as conditions present prior to ALS onset) compared to age-matched controls [37,38]; nonetheless, a substantial portion of ALS patients are treated for antecedent conditions that are common in the age-matched general population, such as hyperlipidemia (e.g., with statins), hypothyroidism (e.g., with levothyroxine), hypertension (e.g., with calcium channel or beta blockers), and diabetes (e.g., with insulin replacements or blood sugar regulators like metformin). Treatment of antecedent conditions has also been controversial in the literature, especially treatment of high cholesterol [39,40] or diabetes, where there is some evidence these treatments could expedite ALS etiology. Likewise, use of pain medications (namely opioids) [41,42] and some categories of antidepressants, which have side effects that induce or exacerbate respiratory depression, are also controversial in ALS patients. Most ALS patients already have compromised respiration due to the paralyzing effects on ALS on the diaphragm and intercostal muscles used for breathing. Thus, even small increases in respiratory depression from palliative treatment could be perceived as a risk [43].

Weighing the potential benefits of improved QoL against the risks of potentially decreasing ALS survival duration is necessary. More analysis is needed to better understand the impact of ALS patient mood and its relationship to physical function and to better understand the quantifiable impact of palliative interventions on ALS survival duration. Discordance among existing studies warrants additional comprehensive evaluations to assess existing hypotheses and to generate new ones. The aims of this study are to: (1) Discern the relationships between physical health, mood, and survival duration in ALS; (2) evaluate the usage of common QoL medications like antidepressants and pain medication on ALS patient mood and survival duration; (3) assess prevalent pharmaceutical and supplement usage to determine potential beneficial or harmful associations with ALS survival duration. A better understanding of these quality of life and survival duration associations can improve ALS clinical care.

## 2. Materials and Methods

This is a secondary analysis of a retrospective cohort study comprising 8028 clinical visit records collected from 1585 patients at the Emory ALS Clinic (Emory University Hospital, Atlanta, GA, United States) into a FileMaker Pro relational database. Data collection, organization, and quality control methods are as previously published in prior work with this data set [5,20,44,45]. All data was de-identified and anonymized. The study was conducted in accordance with the Declaration of Helsinki; the protocol was jointly approved by the Institutional Review Boards of Emory University and Georgia Institute of Technology (Protocol H16257).

### 2.1. Temporal Assessments and Patient Characteristics

Temporal assessments are used to assess associative relationships. ALS start point and end point is used to calculate the most important outcome metric for any ALS assessment, which is survival duration. Survival duration is defined as time passed between ALS start point and end point. ALS end point is defined as the date of death, which is a straightforward determination. ALS start point is defined as the date of initial ALS diagnosis by a physician. 

Patient characteristics assessed as part of this study include gender, ALS onset type, and ALS onset age. Gender is defined as biological gender (male or female). ALS onset type is defined as limb, bulbar, or other/unclassifiable [45]. Table 1 illustrates the patient characteristics of the overall cohort. The majority of the patients resided in the southeast United States.

For the pharmaceutical and supplement portion of this study’s analysis, included patients were further stratified based on their ALS onset age relative to the literature mean of 55 years (e.g., early onset is <55 years and late onset is ≥55 years) and ALS survival duration relative to the pharmaceutical & supplement ALS cohort mean of 3.67 years (e.g., short survival duration is <3.67 years and long survival duration is ≥3.67 years).

### 2.2. Assessment of Clincal Impression of Mood

Initial inclusion criteria for the clinical impression of analysis (CIM) analysis consisted of patients who had a specialist-determined ALS diagnosis and ≥5 clinic visits to see the same ALS clinic specialty team. For each clinic visit, the clinician denoted a short, qualitative description of the patient’s exuded mood, referred to as clinical impression of mood (CIM), as previously published [20]. Text mining of the study database was used to classify clinical impression of mood (CIM) using a binomial score of 0 or 1. A perceived positive or neutral mood for the visit day was assigned a “0”, whereas a perceived negative mood was given a value of “1”. Note that CIM scores for patients with documented pseudobulbar affect (PBA) were excluded because visualized emotion and facial expression of a PBA patients, by clinical definition, does match the patient’s internalized mood during a PBA event.

Text mining automation with a synonym table (Table S1) was utilized to initially and agnostically classify mood according to the previously published method for CIM [20]. However, more ambiguous adjectives (shown in rightmost column of Table S1), required more context to make a determination; for these, three trained experts reviewed the full record before assigning a CIM score for the visit. Individual clinic appointments with missing data or incomplete data on patient mood were excluded from the CIM analysis.

CIM was utilized in the present study due to a lack of standardized quality of life (QoL) surveys for this data set. The advantage of CIM is the lack of patient or caretaker survey bias [20]. The limitation of CIM is the reliance on clinician observed interpretation of verbalized communication or non-verbalized facial expression as an assessment of patient mood.

### 2.3. Assessments of Physical Health and Modulators of Quality of Life

Forty assessments of physical health or modulators of quality of life were classified into the following categories: Respiratory, pain, disability, muscle control, oral muscle control, vocal control, PEG, therapy, QoL medication, depression, sleeping problems, and social. Thirty-four of the assessed factors are of the binary data type, meaning they relate to either a yes or no assessment. For example, a condition is either “present” or “not present”, or a therapy either “used” or “not used” by the patient. Six of the factors are of the continuous data type, meaning they have a quantitative value assigned from a continuous scale. The continuous factors include standard quantitative measures of respiratory function, as well as the revised ALS functional rating scale (ALSFRS-R) [17]. The ALSFRS-R includes two main components, a survey assessment of activities of daily and an assessment of respiratory function; a higher ALSRFRS-R total score or sub-score indicates more function, whereas a lower score correlates with more severe disease progression [17]. The present study uses the ALSFRS-R total score and the ALSFRS-R respiratory sub-score. Most of the assessed physical health factors are standardly measured in ALS, well-defined, and often used by the field in ALS predictive analysis [45].

The categorizations in Table 2 make most of the assessed factors self-explanatory, but a few warrant further explanation. PEG, an acronym for percutaneous endoscopic gastrostomy, is more commonly known as a “feeding tube”; this surgical intervention assists in dysphagia and corresponding weight loss by providing a vehicle for regular nutrition and hydration directly to the stomach. Cough assist and suction are two different interventions to assist with secretion clearance, which is impaired in ALS due to respiratory muscle paralysis. Bi-PAP is an acronym for bi-level positive airway pressure, a form of non-invasive ventilation to improve respiration. Assistive devices include devices that improve activities of daily living such as a wheelchair, walker, leg brace, cane, etc.

Medications that are known to be modulators of quality of life were included in the CIM analysis as binary factors (“use” or “did not use”). As shown in Table 2, these QoL modulators include antidepressant medication usage, drooling medication usage (namely anticholinergics), non-opioid pain medication usage (prescription medication for pain that does not target opioid receptors), opioid pain medication usage (prescription pain medications that target opioid receptors), NSAID medication usage (common over the counter pain medication, like ibuprofen, used to combat pain and inflammation), sleeping medication usage (prescription or over the counter drugs or supplements to induce sleep or treat clinical insomnia), and muscle control medication usage (prescription muscle relaxants or antispastics, like baclofen). Usage of these medications were assessed to determine their potential relationship with CIM. Antidepressant usage was further broken down into sub-categories based on mechanism of action: SNRI, SSRI, tricyclic antidepressant, and general antidepressant.

### 2.4. Other Pharmaceutical and Supplement Usage

A separate analysis was performed to assess all pharmaceutical and supplement medication usage and their associative relationships to ALS onset age, gender, and survival duration. All pharmaceutical or supplements were either prescribed by the ALS clinic or were reported as being used by the patient at their clinic visit (e.g., could be drugs prescribed from another healthcare provider or the patient’s own purchase of over the counter drugs or nutritional supplements). The medications included palliative medications for ALS symptoms, treatments for other antecedent or co-morbid health conditions, and vitamins or nutritional supplements for improved general health or prophylaxis. Medication usage in the present study was evaluated on a per-patient basis. Medications were categorized by pharmacological class, active ingredient, or intended purpose. Based on statistical power for usage sample size (e.g., number of patients using the medication), the top 8.5% of medications were included in the analysis, which equates to 47 medication categories (Table S2).

### 2.5. Data Analysis and Statistical Methods

Four analyses were performed: (1) Analysis to assess CIM and its association to physical health factors; (2) analysis to the association of ALS survival duration with CIM, physical health factors, and known modulators of QoL; (3) analysis to assess the association of CIM with antidepressant usage, including overall antidepressant usage versus non-usage, usage of specific antidepressant sub-classes based on mechanism of action, and number of distinct antidepressants jointly co-used; and 4) analysis to assess the association of the most commonly used pharmaceuticals and supplements in the study cohort with ALS survival duration.

For analyses 1–3 outlined above, a Chi-square test was used to assess correlation between the factors with a binary data type, and a generalized linear model (GLM) was used to assess correlation between CIM and factors with a continuous data type. The GLM type was a binary logistical regression. A standard *t*-test was used to determine if antidepressant usage or non-usage impacted survival duration. A one-way ANOVA analysis was used to determine if a specific antidepressant class had a significant effect on a patient’s CIM. 

For analysis 4, two methods were utilized to assess associations between the usage of a specific medication or supplement category and patient gender, ALS onset age, and survival duration. The Fisher’s exact test was used to determine statistical significance of association of each medication class’s usage with survival duration. Conditional random forests modeling was utilized to assess the importance of pharmaceutical medications and supplements on survival duration. Conditional random forests were chosen over standard random forests to address potential class imbalances in the data [46]. The conditional random forests model was simulated four times and the variable importance was averaged to determine the final result. The area under the curve (AUC) of variable importance was used as the performance metric [45]. 

Confidence intervals of 95% and 99%, respectively, were used for comparison of significance. Associations throughout the results are designated as insignificant (*p*-value > 0.05), low threshold of significance (0.05 > *p*-value ≥ 0.001), or high threshold of significance (*p*-value < 0.001). Recall that significance threshold does not imply differences in the magnitude or strength of the association. Rather, the threshold of significance only represents the probability of a false positive result. All statistical analysis was performed in Matlab™ (the MathWorks, Inc., Natick, MA, USA). 

## 3. Results

The primary goal of this study was to assess associations among patient mood, quality of life modulators, usage of other pharmaceuticals and supplements, and ALS patient survival duration. Mood was assessed using the clinical impression of mood (CIM) metric for each clinic visit [20]. Quality of life modulators included palliative interventions for ALS symptoms; QoL medication usage for depression, pain, and sleep; and factors related to patient social support. Assessment of other pharmaceuticals and supplements examined usage of the most prevalent treatment modalities, regardless of indication, whether for ALS-related symptoms, antecedent disease, co-morbid disease, or general health prophylaxis. Thresholds of statistical significance are denoted for *p* < 0.05 and *p* < 0.001 throughout the results.

### 3.1. Factors Assessed for Association with Clinical Impression of Mood (CIM)

Table 2 lists the 40 health factors analyzed for their correlation with CIM. Eleven of these factors have significant associations with CIM as illustrated in Table 3 where the * indicates a significance threshold where 0.05 > *p*-value ≥ 0.001, and ** denotes a significance threshold where *p*-value < 0.001. The significant factors associated with CIM included both assessment of physical factors as well as QoL modulators.

### 3.2. Physical Health Factors Significantly Associated with Clinical Impression of Mood (CIM) 

Three physical health factors met the high threshold for significance (*p* < 0.001): FVC, % predict FVC, and jaw jerk. FVC and % predict FVC both measure respiratory output. Higher scores correlate to better respiratory function. Thus, unsurprisingly, higher FVC and FVC % predict result in lower CIM (e.g., moods that are happy are neutral). The presence of jaw jerk results in higher CIM (e.g., more depression or anxiety). Other physical health factors that had an association meeting the lower threshold for significance (0.05 > *p*-value ≥ 0.001 included: Toe walk, dysphagia, drooling, ALSFRS-R total score. Ability to do the toe walk represents more physical function and is associated with lower mean CIM. Presence of dysphagia or drooling resulted in a higher mean CIM. A higher ALSFRS-R total score represents a combination of better respiratory function and/or better muscle function to independently perform activities of daily living [17]. A higher ALSFRS-R total score is associated with a more positive mood, equating to a lower mean CIM.

### 3.3. Quality of Life Factors (QoL) Significantly Associated with Clinical Impression of Mood (CIM)

Three quality of life (QoL) factors had associations that met the high threshold for significance (*p* < 0.001): Presence of general pain and the use of therapeutic devices, namely Bi-PAP and cough assist. One QoL factor, no family reported, had an association that met the low threshold for significance (0.05 > *p*-value ≥ 0.001). Not surprisingly, presence of pain is associated in a more negative mood (depression or anxiety), equating to higher mean CIM. Interestingly, use of the Bi-PAP, cough assist intervention was actually associated with a positive mood (happy or neutral), equating to a lower mean CIM.

### 3.4. Physical Health Factors Have Strong Association with Survival Duration

The physical health factors with associations to shorter survival duration that met the high threshold of significance (*p* < 0.001) are: Presence of disability, presence of fasciculation, presence of jaw jerk, presence of dysarthria, lower ALSFRS-R scores (corresponding to less independent function), forced vital capacity (FVC), percent predicted forced vital capacity (FVC % predict), lower ALSFRS-R respiratory sub-score (corresponding to less respiratory function), and negative inspiratory force (NIF). Physical health factors with association to shorter survival duration meeting the low threshold of significance (0.05 > *p*-value ≥ 0.001) are presence of dysphagia, presence of tongue atrophy, and presence of head drop. These results are consistent with other prior studies.

### 3.5. Quality of Life (QoL) Modulators Have Strong Association with Survival Duration

Several quality of life (QoL) modulators had associations with survival duration that met the high threshold for significance (*p* < 0.001): CIM, general pain, cough assist usage, Bi-PAP usage, PEG (“feeding tube”) usage, and not being accompanied to appointment. More specifically, lower CIM (e.g., patients with a more positive or neutral mood), lack of general pain, Bi-PAP usage, PEG usage, cough assist usage, and patients being accompanied to their appointment, are all factors that are individually associated with longer ALS survival duration.

Other QoL factors with associations to longer survival duration that met the lower threshold of significance (0.05 > *p*-value ≥ 0.001) are: Suction usage, lack of reported sleeping problems, reported family support, reported friend support, antidepressant medication usage, sleeping medication usage, muscle related medication usage, and non-opioid pain medication usage. 

Overall, usage of most QoL medications and interventions by ALS patients as well as the general presence of social support was associated with a significant increase in survival duration. The QoL factors in Table 2 that had an insignificant association with survival duration (*p* > 0.05) are drooling medication usage, opioid medication usage, and NSAID medication usage. 

### 3.6. Antidepressant Usage Does not Impact Clincial Impression of Mood (CIM)

Approximately 34% of the patients meeting inclusion criteria for the CIM analysis took one or more antidepressants. Given that antidepressants are prescribed to improve depression or anxiety, it could be hypothesized that antidepressants may have an impact on the clinician-visualized CIM at the clinic visits. To assess this hypothesis, the association between mean CIM score and antidepressant usage was compared. 

**Figure 1:**
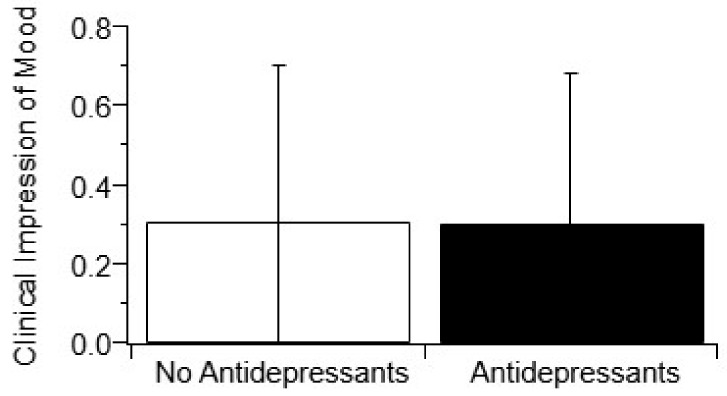
The use of antidepressants does not improve Clinical Impression of Mood (CIM). CIM was classified at each clinic visit using a published binary assessment [20] based on visual and verbal ques, as detailed in the Methods. All antidepressant interventions in a patient’s chart were recorded on a per-visit and per-patient basis. The use of antidepressants (n = 458) did not have a significant effect on a patient’s mood compared to those who did not take antidepressants (n = 717) (*p* > 0.05, *t*-test).

**Figure 2:**
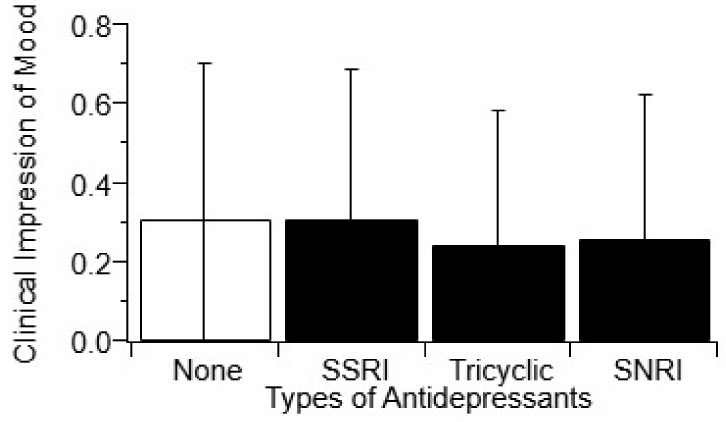
The type of antidepressant does not have an effect on Clinical Impression of Mood (CIM). CIM was classified at each clinic visit using a published binary assessment [20] based on visual and verbal ques as detailed in the Methods. All antidepressant interventions in a patient’s chart were recorded on a per-visit and per-patient basis. Antidepressants were divided into four general categories; SSRIs (n = 348), tricyclic antidepressants (n = 55), SNRIs (n = 56), and general antidepressants. General antidepressants were excluded from this analysis due to the small sample size. The type of antidepressants did not have a significant effect on a patient’s mood compared to those who did not take antidepressants (*p* > 0.05, one-way ANOVA).

“Antidepressant usage” was defined as regular usage of antidepressants over three or more visits where CIM was also measurable. Figure 1 illustrates there is an insignificant change in CIM (*p* > 0.05, *t*-test) with the usage of antidepressants. To assess if antidepressant impact on mean CIM score could be class-specific, antidepressants were divided into their respective pharmacological classes based on mechanisms of action: SSRI, tricyclic, and SNRI. Still, there was no significant association with any class of antidepressant and mean CIM score (Figure 2, *p* > 0.05, one-way ANOVA). Finally, the number of different antidepressants being used by each patient was analyzed. The usage of multiple antidepressant medications also showed no association (*p* > 0.05) with mean CIM (Figure S1).

It should be noted that the deviations shown on Figure 1 and Figure 2 (and Figure S1) for completeness are visually deceiving due to the binary nature of the CIM metric. The statistical variance for all included metrics was actually overall much lower for this large ALS cohort study compared to smaller ALS cohort studies. Nonetheless, there is more variance in mood compared to other physical health metrics. The increased variance in mood is because mood can vary from clinic visit to clinic visit, whereas physical progression in ALS essentially continuously declines with each subsequent clinic visit.

### 3.7. Visualisation of Medication and Supplement Usage Associations

**Figure 3:**
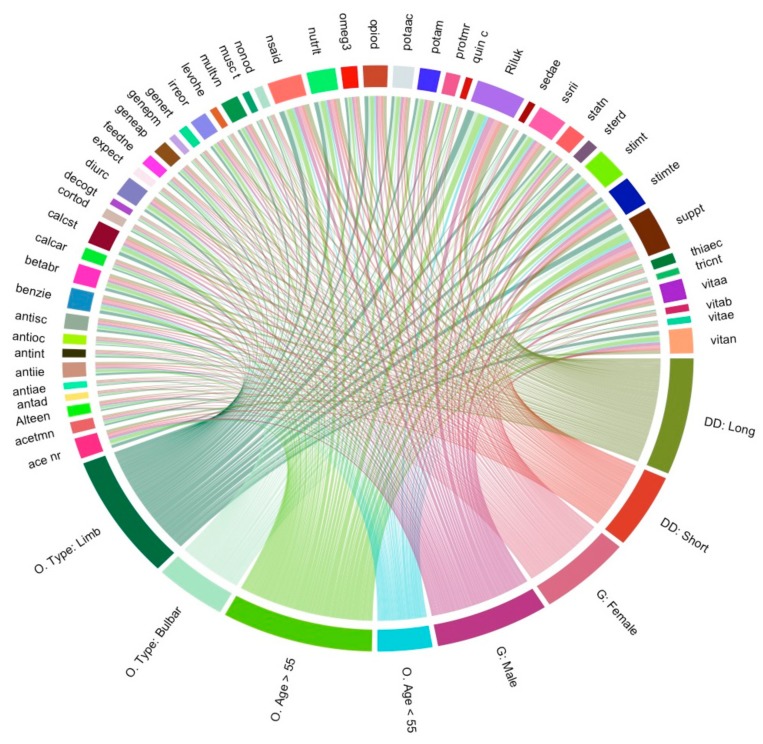
Correlation between the most common disease and intervention categories: “Most common” was defined as usage by more than 8.5% of the ALS cohort (see Methods). On the circular relationship plot, each 1-pixel line illustrates a patient connection. Gender (G) is replaced by “male” or “female”, onset type (O) is replaced with “bulbar” or “limb”, onset age (O. Age) is replaced with “≥55 years” and “<55 years”, disease survival duration (DD) is replaced with “longer” or “shorter” (than the average disease duration, which is equal to 3.67 years for this analysis). Other shorthand symbols are defined in detail in Table S2.

Figure 3 visualizes the covariance and the usage prevalence of each intervention category. Moreover, it specifically shows the connections between intervention usage and ALS patient characteristics (gender, onset age, longer survival duration, shorter survival duration). The shorthand nomenclature for Figure 3 is defined in Table S2. The connections demonstrate the degree that each patient characteristic connects with each intervention. Patients with a longer survival duration have more connections to interventions than those with shorter survival duration. Note that “long” or “short” survival duration is relative to the average survival duration, 3.67 years, for the ALS patients included in the pharmaceutical and supplement cohort analysis. Interestingly, males and females have roughly the same number of intervention connections despite males outnumbering females at an approximate 3:2 ratio. Thus, female patients use more interventions per capita. While a few medications are female-specific, the presence of female-specific interventions (such as progesterone) do not account for the overall higher usage prevalence of multiple simultaneous interventions by females in the patient cohort.

### 3.8. Statistical Assessment of Medication and Supplement Associations 

Patient characteristics and interventions characterized as medications or supplements were used to predict survival duration using both Fisher’s exact test and conditional random forests. For these analyses, non-medication interventions like Bi-PAP usage, cough assist, and social support were not included. 

First, Fisher’s exact test was used to determine the significance of the association of medication usage with survival duration. Of the 47 medication and supplement categories (see Table S2 for detailed definitions), 17 were associated with a significantly longer increase in survival duration (*p* < 0.05) ranging from +4.5 months to +16.5 months. No oral medication or supplement was found to result in a significant decrease in survival duration. Three medications had associations with longer survival duration meeting the high level significance threshold (*p* < 0.001): Alternative herbal medicines (+9.6 months), Vitamin A (+8.9 months), and multivitamins (+8.2 months). Table 4 illustrates the 17 medication or supplement categories with significant associations (*p* < 0.05) with longer survival duration, and Table S3 illustrates the 30 medication or supplement categories, which had insignificant associations with survival duration.

Of the 17 interventions associated with longer survival duration, only five interventions were found to have a significantly higher usage prevalence (*p* < 0.05) on the basis of onset age; all five interventions (Vitamin A, muscle relaxants, antispastics, [non-specific] vitamin, and anticholinergics) were more prevalently utilized by the younger onset age group (onset age < 55 years). Seven of the 17 interventions that statistically increased survival duration had a statistically higher usage prevalence (*p* < 0.05) on the basis of gender; all nine categories (stimulant, stimulant laxative, nutritional supplement, antihistamine, [non-specific] vitamin, non-opioids, anticholinergics, and muscle relaxants) were more prevalently utilized by females than males. Finally, four of the 17 interventions that statistically increased survival duration had a statistically higher usage prevalence (*p* < 0.05) on the basis of onset type. Four interventions were more prevalently used by limb onset (muscle relaxants, antispastics, vitamin A, [non-specific] vitamin), whereas one intervention was more prevalently used by bulbar onset (anticholinergics). 

The Fisher’s exact test alone, does not account for complex interactions that influence survival duration. However, random forests do include complex interactions between all variables when determining which are most predictive of the response (e.g., survival duration). Note that, while other non-drug interventions were excluded in the random forests analysis, the feeding tube was included in the random forests because it corresponded to supplement usage. Nonetheless, feeding tube supplement usage was separated from the nutritional supplement category, the latter which encompassed prepared meals or canned nutrition drinks.

**Figure 4:**
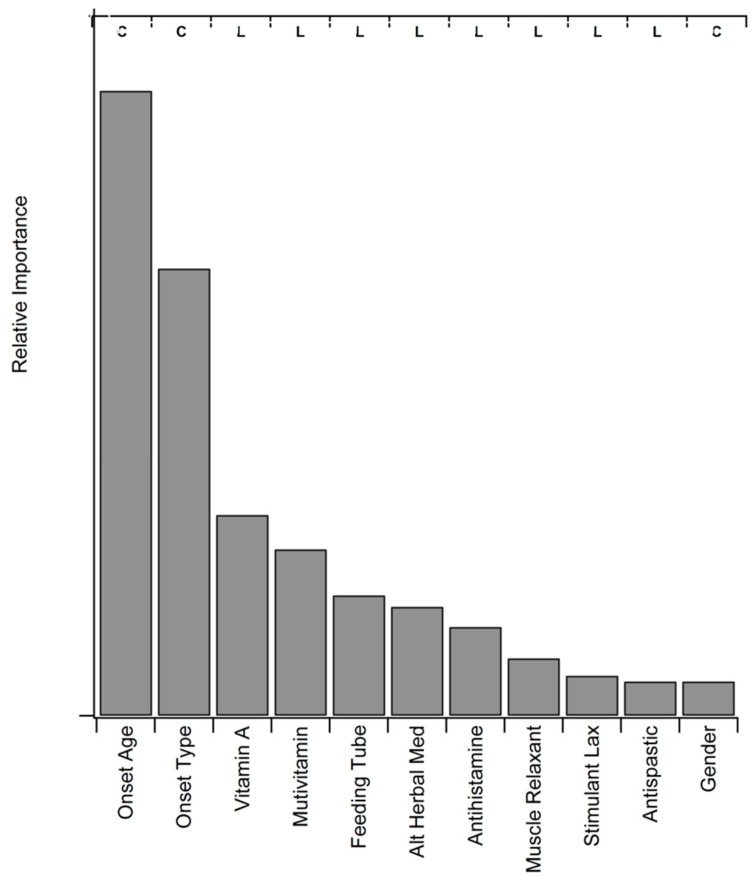
Pharmaceuticals & supplements and patient characteristics as predictors of ALS disease duration. Conditional random forests were used to determine how much each individual metric helped increase the area under the curve (AUC), the primary metric of model performance prediction. Relative importance is normalized and scaled for ease of visualization. Note that “feeding tube” represents nutritional supplements given via a percutaneous endoscopic gastrostomy (PEG).”C” denotes that the factor is a patient characteristic versus a medication, and “L” represents that the medication or supplement is more strongly associated with longer survival duration.

Figure 4 illustrates the relative area under the curve (AUC) performance for variables that best predict survival duration using conditional random forests modeling. The “best” predictive variables included three patient characteristics (ALS onset age, ALS onset type, and gender) and eight medication & supplement categories. The collinear onset age and onset type are, unsurprisingly, the top two predictors whereas gender, the least predictive of the patient characteristics, was ranked 11th. The eight in-between predictors of disease duration, respectively ranking 3 through 10, are all medication or supplement categories: Vitamin A, multivitamin, feeding tube supplements, alternative herbal medicine, antihistamine, muscle relaxant, stimulant laxative, and antispastics. All eight medications & supplements have a higher usage prevalence in the longer disease duration population. Notably, the eight medications & supplements predicted as most important from the conditional random forests were also predicted to be significant at the high threshold (*p* < 0.001) in the Fisher’s exact test. 

## 4. Discussion

There is a delicate balance between increasing patient quality of life and extending patient life span. The large sample size of the present study (a retrospective cohort of 1585 patients) provides more clarity on the controversial findings seen in the literature for the associations of patient mood, modulators of quality of life (QoL), and usage of pharmaceuticals and supplements with ALS patient survival duration. The key findings include: (1) Patient mood as assessed by the clinical impression of mood (CIM) metric has a strong relationship to both physical health factors and modulators of QoL; (2) antidepressant usage in the present ALS cohort had no impact on CIM; (3) CIM, modulators of QoL (including both social support factors and palliative interventions used to treat ALS and related symptoms), and several other pharmaceutical drugs and supplements, had significant positive associations with extending survival duration. The details of these conclusions are discussed below in context with other literature findings.

### 4.1. Mood and Social Support are Associated with Increased Survival Duration

An important component in QoL is the patient’s mood throughout the course of the disease. The will to live has been shown to be a crucial factor in increasing survival duration in many intractable or terminal diseases [7]. Additionally, there is evidence that higher QoL improves survival in patients with terminal prognoses [47,48]. In the present study, a more positive mood (happy or neutral), as measured via CIM, was strongly correlated with an increase in survival duration (*p* < 0.001).

An important aspect of QoL is social support by humans that interact with patients on a regular basis and/or accompany patients to their ALS clinic appointments. Patients being accompanied to their appointment met the high significance threshold (*p* < 0.001) for association with a more positive mood, as measured by CIM. Furthermore, all of the factors characterized as social support were significant (*p* < 0.05) for increasing survival duration: Reported family support, reported friend support, and patient being accompanied to clinic appointment. 

### 4.2. Palliative ALS Interventions Associated with Improved Mood and Survival Duration

When comparing to prior work [49,50], it was not surprising that mood (measured via CIM) strongly correlated with physical health factors like respiratory metrics (i.e., FVC % predict, FVC, NIF, ALSFRS-R score, etc.) [5,45]. Moreover, existing studies that show respiratory interventions improve survival duration [51,52], although “mood” was not specifically assessed in prior work. Thus, the finding that certain palliative interventions that improve respiration or secretion clearance, namely bi-level positive airway pressure (Bi-PAP) and cough assist, are associated with a more positive (happy or neutral) mood is novel. Both Bi-PAP and cough assist usage met the high threshold for significant association with a more positive mood, as measured via mean CIM (*p* < 0.001, Table 3).

Some studies have pointed out that Bi-PAP usage compliance can be difficult in a segment of patients because it requires wearing a mask attached to a non-portable compressor plugged into an electrical outlet [5,45]. Depending on individualized respiratory function, patients must use Bi-PAP for prolonged periods of time, ranging from 8 hours/day (overnight while sleeping) up to 24 hours/day. Thus, while Bi-PAP usage is known to improve respiration and survival duration [5], it nonetheless can also reduce overall patient comfort, especially while sleeping; impede the patient’s ability to communicate; and impede patient mobility. The novel result that Bi-PAP usage is associated with a more positive mood is important for prescribers and patients alike. That is, the benefits of Bi-PAP usage, both in associatively increasing patient survival and in modulating better patient moods, appear to outweigh the perceived QoL negatives. 

Cough assist is used intermittently several times a day to help patients clear secretions. A mask is put on temporarily (for seconds to minutes) and cycles of positive and negative pressure are applied to trigger a cough reflex, which helps to clear secretions. There are not as many perceived negative side effects of the intervention simply because its usage is not prolonged. However, fear of strangling on secretions is a perceived quality of life negative that could impact patient mood. Nonetheless, cough assist usage was actually significantly associated with a more positive mood as measured via mean CIM score. 

Recent findings showed there is much synergy when using Bi-PAP and cough assist, which greatly increases survival duration compared to using either intervention alone (e.g., up to a +15 month increase in survival) [5]. The present study’s finding of a significant association between a positive patient mood and using cough assist or Bi-PAP amplifies the overall importance of these interventions by diminishing fear related to perceived negative quality of life effects.

Interestingly, there is no significant association of the usage of percutaneous endoscopic gastrostomy (PEG), more commonly known as a “feeding tube”, with mood. Qualitative assessment of the trend also indicates no negative tendency towards lower mood. The initial placement and upkeep of the PEG intervention is much more invasive and risky than Bi-PAP or cough assist. While no relationship was found with mood, there was a very strong association with PEG nutritional supplements and increased survival duration in the Chi-square test and the conditional random forests (Figure 4) conducted in the present study. The significance of PEG usage in associatively increasing survival is corroborated by recent work [20,53].

### 4.3. Antidepressant, Pain, Sleeping Medication Correlate with Survival Duration but not Mood

Although ALS mostly effects motor versus sensory neurons, many patients report increased pain, which is likely exacerbated by spasticity or inability to move, resulting in stiffness, muscular, and joint pain. In the present study, presence of general pain did significantly (*p* < 0.001) correlate with a more negative patient mood (Table 3). Yet, usage of any of the pain medication types shown in Table 2 (opioids, non-opioids, or NSAIDs) did not correlate with improved CIM. This is interesting, especially the lack of correlation with opioids, which are generally thought to modulate reward pathways that impact mood [54]. Non-opioids outperform opioids in terms of association with survival duration. Non-opioids had a statistically significant associative increase in survival duration (*p* < 0.05, +16.5 months) whereas opioids had only a qualitative, insignificant association with survival duration (*p* > 0.05, +6.5 months).

Antidepressants are used to combat depression, whether it be directly tied to frontotemporal dementia or other cognitive decline that occurs in about 25% of patients [55], or for depression simply related to the psychological and emotional weight of the diagnosis, corresponding symptoms, and fear of death [7,8]. More simply stated, antidepressants are usually prescribed to help improve patient mood, namely depression and anxiety. Yet, antidepressant usage, whether overall usage versus non-usage, usage of a specific class of antidepressants, or the number of different antidepressants used, had no significant impact on patient mood as measured by mean CIM (Figure 1 and Figure 2, and Figure S1). The finding that there is a lack of efficacy in antidepressants for changing ALS patient depression is not unprecedented [56,57]. Despite the fact that antidepressants did not associate with CIM, one class did significantly (*p* < 0.05) and positively associate with longer disease duration, tricyclic antidepressants. In fact, tricycle antidepressants had one of the largest changes in survival duration (+16.4 months) as shown in Table 4.

The present study found “general pain” was associated with poorer patient mood and shorter survival duration (*p* < 0.001). Others have also pointed out that pain management is a critical aspect of ALS patient care and quality of life [58]. Nonetheless, a common concern, especially with opioids and to a lesser extent some antidepressants, is respiratory depression. Specifically, in ALS, opioids could have an undesired synergistic effect that exacerbates respiratory depression in ALS patients that either already have or are susceptible to respiratory muscle weakness. Yet, tricyclic antidepressants and opioids had individual associative increases in survival duration of +16.4 months and +15.2 months, respectively. These associative relationships are certainly not 1:1 with survival duration; evidence to support this conclusion is seen in the random forests, which did not pick either tricyclic antidepressants or non-opioids in the top 11 predictors of survival duration. Nonetheless, their significance in the Fisher’s exact test and the sheer magnitude of their associative survival duration changes, which ranked first and third overall, does warrant additional investigation.

General sleep (as in patient-reported problems with sleep, namely insomnia) did not significantly impact patient mood as measured via mean CIM. Likewise, use of sleeping medications had no association with mean CIM. However, the “general sleep” category of assessed pharmaceuticals and supplements did show a significant association (*p* < 0.05) with survival duration in the Fisher’s exact test. ALS patients who used general sleep medications had a +4.9 month associative increase in survival duration (Table 4). This category included patients who took over the counter, natural melatonin supplements. Melatonin, which also happens to be an antioxidant, was found to be helpful in ALS mice, and not harmful in humans [59]. It is possible that the positive association of sleep medications on ALS survival duration is not tied to quality of life, but rather other etiological aspects, such as providing a better sleep state for the body to repair, whether through combatting oxidative stress [60] or another mechanism. Again, further investigation is warranted.

### 4.4. Pharmaceuticals for ALS-Related Muscle Spasm and Fasiculation Symptoms

Muscle spasticity and fasciculation have been shown to correlate with survival duration in prior work examining varied ALS cohorts [61,62]. Here, presence of fasciculation was not significantly correlated with patient mood, but met the high threshold for significance (*p* < 0.0001) for association with decreased survival duration. Muscle relaxants and antispastic drugs are the two classes of pharmaceuticals most utilized to treat these ALS-related symptoms. Usage of muscle relaxants or antispastic drugs was associated with a significant (*p* < 0.05) increase in survival duration of +13.4 and +16.5 months, respectively. Both classes of these drugs met the high threshold of significance (*p* < 0.001) for associations to the younger ALS onset age (ALS onset < 55 years) and the longer survival duration (>3.67 years) sub-populations. Both muscle relaxants and antispastic drugs also were selected in the top 11 predictors for survival duration by the conditional random forests (Figure 4).

### 4.5. Pharmaceuticals for ALS-Related Secretion Clearance Dysfunction

As noted earlier, mechanical interventions like Bi-PAP, cough assist, and suction are used to improve overall respiration and/or improve secretion clearance. However, there are also other prescribed pharmaceuticals, namely for drooling. Anti-drooling medications (primarily anticholinergics) are significantly associated with a longer disease duration in both bulbar and limb patients (+6.2 months, *p* < 0.05). Decreasing excessive salivary secretion not only improves quality of life, but also decreases risks of associated aspiration in the presence of dysphagia. Of note is that only one anti-drooling medication, glycopyrrolate, was found to be less impactful on survival duration, but this could have been due to sample size. Markedly, almost half of the patients taking anti-drooling interventions also take antidepressants. This statistic suggests drooling and depression are linked, which has also been shown in prior studies [49,50]; this hypothesis is corroborated in the present study in the Chi-square analysis, which identified a significant association (*p* < 0.05) between drooling and a more negative (e.g., depressed or anxious) mood as measured by mean CIM. Notably, the patient sub-population using both anti-drooling and anti-depressant interventions in combination had a longer disease duration than sub-populations using either intervention alone. 

Finally, antihistamines, which have not been greatly studied in ALS, appear to be associatively beneficial in extending disease duration in the present study (+8.4 months, *p* < 0.05). Moreover, antihistamines ranked as the 5th best intervention in predicting disease duration according to the random forests, which accounts for complex interactions. It is hypothesized that the efficacy of antihistamines is related to airway clearance and enabling easier respiration [63].

### 4.6. “Controversial” Interventions that May be Related to Decreased Survival Duration

There are some interventions that have been shown in previous studies to be contraindicated in ALS due to their relationship with shorter survival duration, namely medications used to treat diabetes [64] and hyperlipidemia (high cholesterol) [39,40]. Additionally, hypertension (high blood pressure) is sometimes an issue, not only as an antecedent condition, but as an attribute of ALS etiology and riluzole treatment [65,66]. No medication examined in the present study was found to significantly decrease disease duration in the Fisher’s test, but a few related to these antecedent or co-morbid conditions did show qualitative trends towards decreased survival duration. Metformin, a type 2 diabetes medication that is also used to treat insulin resistance, appeared to only slightly decrease disease duration, albeit not significantly (*p* > 0.05). Statins were associated with an insignificant (*p* > 0.05) decrease in survival duration (−3.7 months) in the Fisher’s test. Statins, in particular, have been more debated in the ALS literature, as other studies have found usage may decrease survival duration, possibly through an etiological mechanism [39,40]. Finally, the thiazide diuretic (treatment for hypertension or to maintain water balance) also had a qualitative decrease in change in survival duration (−2.4 months) that was also insignificant in this cohort (*p* > 0.05). Additional research with even larger samples sizes is necessary to determine with certainty whether the above “controversial” interventions do in fact decrease survival duration.

It appears that these perceived negative relationships are intertwined with ALS onset age. These antecedent conditions and their treatment are more prevalent in the later ALS onset (≥55 years of age at ALS diagnosis) sub-population. Interestingly, prior work has illustrated that antecedent disease could be associated with potential neuroprotection, which results in delayed ALS onset [37,38]. Thus, this could explain why some medications for antecedent disease, namely hyperlipidemia and diabetes, are not beneficial, while other pharmaceuticals like antihistamines, which are lesser studied with no known etiological impact on ALS, appear to significantly increase survival. Antecedent disease and the use or non-use of pharmaceuticals and supplements that have widespread systemic interactions could be further modulating complex, multi-factorial ALS etiology. In particular, the liver has been shown in multiple studies to be a potential effector of ALS etiology. Ironically, ALS patients have less overall overt liver disease, and some liver medications have shown positive impacts in ALS [37]. Moreover, the liver is critical for processing drugs and supplements, and plays a critical role in overall homeostasis [67], which is also known to be greatly impacted by ALS, as well as biological aging.

Finally, some contraindications and even discourse in the literature could be attributed to changes in the activation of the anti-aging gene. Recent studies [68,69] have identified molecular underpinnings consistent with a substantial portion of this study’s results. For example, SIRT1 is an NAD+-dependent deacetylase that functions in a variety of cells and tissues to mitigate age-associated diseases [68]. However, it remains unknown if SIRT1 also acts to prevent pathological changes that accrue in motor neurons during aging and ALS [68].

## 5. Conclusions

The results of this large, retrospective cohort study illustrate the significant associations of patient mood, modulators of quality life, and multiple non-ALS specific pharmaceuticals and supplements on ALS patient survival duration. While there is not yet a cure for ALS, this study illustrates multiple practices that can improve ALS patient quality of life and corresponding survival duration.

The results of this study underscore the importance of patient mood and its significant relationship to survival duration. Not surprisingly, measures of ALS progression do significantly contribute to worse patient mood. However, presence of pain and lack of social support also significantly contribute to worse patient mood. Being accompanied to a clinic appointment and the presence of family or friend support were found to be critical for significantly improving patient mood; this type of social support could be increased through ALS social programs and/or better awareness among families and caretakers on the importance of social support in patient outcome. Interestingly, the use of antidepressants appears to have no effect on ALS patient mood in this large cohort study. This could be due to changes in the etiology underlying depression in the ALS neuropathology, or it could be that the sources of poor ALS patient mood are not readily treatable with antidepressants.

The results of this study identify several therapies, pharmaceuticals, and supplements with significantly positive association with survival duration, which warrant changes in clinical practice. The novel finding that significant survival-enhancing respiratory interventions (Bi-PAP and cough assist) are also associated with a significantly overall better patient mood should greatly increase the prevalence of physician prescription and patient usage of these therapies. Additionally, the negative association of pain with ALS patient mood and the large, positive association of pain medication usage with survival duration, especially non-opioids, indicates pain management should be more at the forefront of ALS patient care. Fasciculation and muscle spasms are typically considered only palliative symptoms, but could have a more complex role in ALS outcomes; both antispastics and muscle relaxant usage was significantly correlated with longer survival in all analyses, whereas the presence of untreated fasciculation and spasms correlated with shorter survival. Finally, the results of this study illustrates that there should be more emphasis on ALS patient health prophylaxis with nutritional supplements, vitamins, and alternative herbs, as they strongly correlated with longer survival duration. Feeding tube usage (PEG) also increased survival duration and did not negatively impact patient mood in the present cohort.

There were a few interesting results that do not yet implore clinical recommendation but do implore more investigation. Specifically, it is interesting that antihistamines and tricyclic antidepressants significantly increased survival duration, ranking among the highest in all analyses, without a clear connection to ALS symptoms or etiology. Additionally, the potential contraindication of diuretics, antihypertensives, diabetic drugs, and hyperlipidemia drugs is supported by insignificant but qualitative associations with decreased survival duration. The complex interactions of antecedent disease and the treatment of antecedent disease with ALS onset and progression warrants further study. Finally, the results of this study illustrate that additional investigation of “off label” drugs for synergistic benefits in ALS could further improve ALS patient quality of life while we await a cure.

## Figures and Tables

**Table 1:** Overall cohort characteristics for biological gender, race, ALS onset type, and ALS onset age.

N = 1585 Patients	
Gender	N (%)
Male	945 (59.62)
Female	640 (40.38)
**Race**	
Caucasian	923 (58.23)
African American	196 (12.37)
Hispanic/Latino	19 (1.20)
Asian	17 (1.07)
Native American	1 (0.06)
Mixed/Other	12 (0.76)
Unspecified	417 (26.31)
**ALS Onset Type**	
Limb	1098 (69.27)
Bulbar	428 (27.00)
Other/unclassifiable	59 (3.72)
**ALS Onset Age**	
<55 years	509 (32.11)
≥55 years	602 (67.89)

**Table 2:** Categories of physical health and intervention usage factors utilized to examine associations with clinical impression of mood (CIM).

Category	Assessed Factors
Respiratory	forced vital capacity (FVC), percent predicted FVC (% predict), negative inspiratory force (NIF), oxygen saturation, ALSRFRS-R respiratory sub-score
Pain	general pain
Disability	disability present, ALRFRS-R total score, paraplegia, quadriplegia, hemiparesis
Muscle Control	head drop, jaw jerk, toe walk, atrophy, fasciculation
Oral Muscle Control	drooling, tongue atrophy, tongue fasciculation, dysphagia
Vocal Control	dysarthria, dysphasia
PEG tube	regular use of surgically inserted PEG tube for nutrition and/or hydration
Therapy	assistive device usage, cough assist usage, suction usage, Bi-PAP usage
QoL Medication	antidepressant usage, drooling medication usage, non-opioid pain usage, opioid pain usage, NSAID usage, sleeping medication usage, muscle-related medication usage
Depression	depression reported
Social	accompaniment to appointment, family or friend support, hospice care, issues in home reported, reported changes in behavior
Sleep	reported sleeping problems

For the purpose of the CIM association analysis, all assessed factors are binary (present or not present; using or not using, etc.) except for five continuous metrics, which include forced vital capacity (FVC); percent predicted FVC (% predict FVC); negative inspiratory force (NIF); oxygen saturation, ALSFRS-R respiratory sub-score, and ALSFRS-R total score.

**Table 3:** Factors with a significant association with clinical impression of mood (CIM).

Factor	N	*p*-Value	Relationship to CIM
Cough assist	1484	**	Users (+) = (↓CIM)
Bi-pap usage	1979	**	Users (+) = (↓CIM)
Jaw jerk	2112	**	↑jaw jerk = (↑CIM)
Toe walk	142	*	↑toe walk = (↓CIM)
Dysphagia	840	*	dysphagia (+) = (↑CIM)
Drooling	2702	*	drooling (+) = (↑CIM)
General pain	671	**	pain (+) = (↑CIM)
No family reported	4175	*	No family reported (+) = (↑CIM)
ALSFRS-R total	848	*	↑ALSFRS-R = (↓CIM)
FVC percent predict	1300	**	↑FVC %predict = (↓CIM)
Forced vital capacity (FVC)	1272	**	↑FVC = (↓CIM)

Note: Clinic visit sample size, N, denotes number of visits where both the listed factor and CIM were assessed within the same clinic visit. Factors shown all have a significant association with CIM where * indicates significance threshold where 0.05 > *p*-value ≥ 0.001, and ** denotes significance threshold where *p*-value < 0.001. CIM is assigned for each clinic visit as binary value with “0” indicating a positive mood (e.g., happy or neutral), whereas “1” indicates a negative mood (e.g., depression or anxiety). The direction of the significant relationship with CIM is indicated by the up (↑) and down (↓) arrows. ↓CIM means the association is associated with a positive mood (e.g., happy or neutral), whereas ↑CIM is associated with a negative mood (depression or anxiety).

**Table 4:** Pharmaceutical medications and supplements with a significant association with longer survival duration.

Category	F User Ratio	M User Ratio	Gender *p*-Value	Bulbar User Ratio	Limb User Ratio	User Onset *p*-Value	User Age (yrs)	Age *p*-Value	Short Dur Ratio	Long Dur Ratio	Surv Dur *p*-Value	△ Surv (mo.)
alternative herb med	0.14	0.17		0.17	0.15		59.4		0.11	0.21	**	9.7
vitamin a	0.31	0.29		0.25	0.32	*	60.1		0.22	0.33	**	8.9
multivitamin	0.23	0.22		0.20	0.24		60.3		0.18	0.28	**	8.2
muscle relaxant	0.13	0.10	*	0.06	0.13	**	56.0	**	0.07	0.13	*	13.4
antispastic	0.23	0.21		0.16	0.24	**	54.5	**	0.17	0.25	*	16.5
vitamin	0.38	0.34	*	0.31	0.38	*	60.4		0.29	0.39	*	6.2
stimulant	0.45	0.38	**	0.40	0.42		59.6		0.37	0.46	*	7.7
sedative	0.09	0.08		0.08	0.09		56.4	**	0.07	0.12	*	4.6
stimulant laxative	0.45	0.37	**	0.40	0.42		59.7		0.36	0.45	*	7.1
antihistamine	0.23	0.18	*	0.20	0.20		59.7		0.17	0.24	*	8.4
vitamin e	0.07	0.08		0.07	0.09		57.2	*	0.07	0.11	*	12.4
nutritional supplement	0.41	0.37	*	0.39	0.40		59.4	*	0.32	0.40	*	9.7
tricyclic anti-depressant	0.09	0.07		0.10	0.07		56.8	*	0.07	0.11	*	16.4
non-opioid	0.14	0.10	*	0.10	0.13		59.3		0.09	0.14	*	15.3
anticonvulsant	0.15	0.12		0.11	0.14		58.8		0.10	0.14	*	8.7
general sleep	0.22	0.19		0.21	0.20		60.7		0.16	0.21	*	4.9
anticholinergic	0.15	0.11	*	0.27	0.07	**	61.3		0.12	0.16	*	6.2

Each pharmaceutical and supplement category is assessed for an association with gender (F for female or M for male), mean user onset age (in years), and survival duration (“short dur” is for short survival duration, “long dur” is for long survival duration, and “Δ surv” is change in survival duration in months). The p-values symbols indicate significance threshold where * denotes low significance threshold of 0.05 > *p*-value ≥ 0.001 and ** denotes high significance threshold where *p*-value < 0.001. The ratio examines the ratio of users to non-users with a specific characteristic as labeled in the column header.

## Supplementary Materials

The following are available online at https://www.mdpi.com/2076-328X/10/1/33/s1, Table S1: Clinical impression of mood (CIM) keyword scoring table. Table S2: Definitions, sample size, and key for the top 8.5% of other pharmaceuticals and supplements. Table S3: Prevalent pharmaceuticals and supplements, which did not have a significant association with survival duration. Figure S1: The Clinical Impression of Mood (CIM) does not have an effect on the number of antidepressants taken by ALS patients.

## References

[1] Zarei S., Carr K., Reiley L., Diaz K., Guerra O., Altamirano P.F., Pagani W., Lodin D., Orozco G., Chinea A. (2015). A comprehensive review of amyotrophic lateral sclerosis. Surg. Neurol. Int..

[2] Petrov D., Mansfield C., Moussy A., Hermine O. (2017). ALS Clinical Trials Review: 20 Years of Failure. Are We Any Closer to Registering a New Treatment?. Front. Aging Neurosci..

[3] National Cancer Institute at the National Institutes of Health NCI Dictionary of Cancer Terms. https://www.cancer.gov/publications/dictionaries/cancer-terms/def/quality-of-life.

[4] Lee M.K., Baek S.K., Kim S.Y., Heo D.S., Yun Y.H., Park S.R., Kim J.S. (2013). Awareness of incurable cancer status and health-related quality of life among advanced cancer patients: A prospective cohort study. Palliat. Med..

[5] Khamankar N., Coan G., Weaver B., Mitchell C.S. (2018). Associative Increases in Amyotrophic Lateral Sclerosis Survival Duration With Non-invasive Ventilation Initiation and Usage Protocols. Front. Neurol..

[6] Majmudar S., Wu J., Paganoni S. (2014). Rehabilitation in amyotrophic lateral sclerosis: Why it matters. Muscle Nerve.

[7] Johnston M., Earll L., Giles M., McClenahan R., Stevens D., Morrison V. (1999). Mood as a predictor of disability and survival in patients newly diagnosed with ALS MND. Br. J. Health Psychol..

[8] Van Groenestijn A.C., Kruitwagen-van Reenen E.T., Visser-Meily J.M., van den Berg L.H., Schroder C.D. (2016). Associations between psychological factors and health-related quality of life and global quality of life in patients with ALS: A systematic review. Health Qual. Life Outcomes.

[9] Chio A., Logroscino G., Hardiman O., Swingler R., Mitchell D., Beghi E., Traynor B.G., Eurals C. (2009). Prognostic factors in ALS: A critical review. Amyotroph. Lateral Scler..

[10] Lou J.S., Moore D., Gordon P.H., Miller R. (2010). Correlates of quality of life in ALS: Lessons from the minocycline study. Amyotroph. Lateral Scler..

[11] Simmons Z. (2015). Patient-Perceived Outcomes and Quality of Life in ALS. Neurotherapeutics.

[12] Bourke S.C., McColl E., Shaw P.J., Gibson G.J. (2004). Validation of quality of life instruments in ALS. Amyotroph. Lateral Scler. Other Mot. Neuron Disord..

[13] Cella D., Nowinski C., Peterman A., Victorson D., Miller D., Lai J.S., Moy C. (2011). The Neurology Quality-of-Life Measurement Initiative. Arch. Phys. Med. Rehabil..

[14] Clarke S., Hickey A., O’Boyle C., Hardiman O. (2001). Assessing individual quality of life in amyotrophic lateral sclerosis. Qual. Life Res..

[15] Epton J., Harris R., Jenkinson C. (2009). Quality of life in amyotrophic lateral sclerosis/motor neuron disease: A structured review. Amyotroph. Lateral Scler..

[16] Felgoise S.H., Stewart J.L., Bremer B.A., Walsh S.M., Bromberg M.B., Simmons Z. (2009). The SEIQoL-DW for assessing quality of life in ALS: Strengths and limitations. Amyotroph. Lateral Scler..

[17] Cedarbaum J.M., Stambler N., Malta E., Fuller C., Hilt D., Thurmond B., Nakanishi A. (1999). The ALSFRS-R: A revised ALS functional rating scale that incorporates assessments of respiratory function. BDNF ALS Study Group (Phase III). J. Neurol. Sci..

[18] Jenkinson C., Fitzpatrick R., Brennan C., Swash M. (1999). Evidence for the validity and reliability of the ALS assessment questionnaire: The ALSAQ-40. Amyotroph. Lateral Scler. Other Mot. Neuron Disord..

[19] O’Boyle C.A. (1997). Quality of Life assessment: A paradigm shift in healthcare?. Ir. J. Psychol..

[20] Bond L., Ganguly P., Khamankar N., Mallet N., Bowen G., Green B., Mitchell C.S. (2019). A Comprehensive Examination of Percutaneous Endoscopic Gastrostomy and Its Association with Amyotrophic Lateral Sclerosis Patient Outcomes. Brain Sci..

[21] Carr A.J., Higginson I.J. (2001). Measuring quality of life—Are quality of life measures patient centred?. Br. Med. J..

[22] Chio A., Gauthier A., Montuschi A., Calvo A., Di Vito N., Ghiglione P., Mutani R. (2004). A cross sectional study on determinants of quality of life in ALS. J. Neurol. Neurosurg. Psychiatry.

[23] Leigh P.N., Swash M., Iwasaki Y., Ludolph A., Meininger V., Miller R.G., Mitsumoto H., Shaw P., Tashiro K., Van Den Berg L. (2004). Amyotrophic lateral sclerosis: A consensus viewpoint on designing and implementing a clinical trial. Amyotroph. Lateral Scler..

[24] Mitsumoto H., Del Bene M. (2000). Improving the quality of life for people with ALS: The challenge ahead. Amyotroph. Lateral Scler. Other Mot. Neuron Disord..

[25] Robbins R.A., Simmons Z., Bremer B.A., Walsh S.M., Fischer S. (2001). Quality of life in ALS is maintained as physical function declines. Neurology.

[26] Mundt J.C., Vogel A.P., Feltner D.E., Lenderking W.R. (2012). Vocal acoustic biomarkers of depression severity and treatment response. Biol. Psychiatry.

[27] Williamson J.R., Quatieri T.F., Helfer B.S., Horwitz R., Yu B., Mehta D.D. (2013). Vocal biomarkers of depression based on motor incoordination. Proceedings of the 3rd ACM International Workshop on Audio/Visual Emotion Challenge.

[28] Kim S.Y., Kim J.M., Kim S.W., Shin I.S., Bae K.Y., Shim H.J., Hwang J.E., Bae W.K., Cho S.H., Chung I.J. (2013). Does awareness of terminal status influence survival and quality of life in terminally ill cancer patients?. Psychooncology.

[29] Averill A.J., Kasarskis E.J., Segerstrom S.C. (2007). Psychological health in patients with amyotrophic lateral sclerosis. Amyotroph. Lateral Scler..

[30] Cupp J., Simmons Z., Berg A., Felgoise S.H., Walsh S.M., Stephens H.E. (2011). Psychological health in patients with ALS is maintained as physical function declines. Amyotroph. Lateral Scler..

[31] Koschnitzky J.E., Quinlan K.A., Lukas T.J., Kajtaz E., Kocevar E.J., Mayers W.F., Siddique T., Heckman C.J. (2014). Effect of fluoxetine on disease progression in a mouse model of ALS. J. Neurophysiol..

[32] Peng Q., Masuda N., Jiang M., Li Q., Zhao M., Ross C.A., Duan W. (2008). The antidepressant sertraline improves the phenotype, promotes neurogenesis and increases BDNF levels in the R6/2 Huntington’s disease mouse model. Exp. Neurol..

[33] Wang H., Guan Y., Wang X., Smith K., Cormier K., Zhu S., Stavrovskaya I.G., Huo C., Ferrante R.J., Kristal B.S. (2007). Nortriptyline delays disease onset in models of chronic neurodegeneration. Eur. J. Neurosci..

[34] Taler M., Miron O., Gil-Ad I., Weizman A. (2013). Neuroprotective and procognitive effects of sertraline: In vitro and in vivo studies. Neurosci. Lett..

[35] Then C.K., Liu K.H., Liao M.H., Chung K.H., Wang J.Y., Shen S.C. (2017). Antidepressants, sertraline and paroxetine, increase calcium influx and induce mitochondrial damage-mediated apoptosis of astrocytes. Oncotarget.

[36] Then C.K., Chi N.F., Chung K.H., Kuo L., Liu K.H., Hu C.J., Shen S.C., Lin Y.K. (2017). Risk analysis of use of different classes of antidepressants on subsequent dementia: A nationwide cohort study in Taiwan. PLoS ONE.

[37] Hollinger S.K., Okosun I.S., Mitchell C.S. (2016). Antecedent Disease and Amyotrophic Lateral Sclerosis: What Is Protecting Whom?. Front. Neurol..

[38] Mitchell C.S., Hollinger S.K., Goswami S.D., Polak M.A., Lee R.H., Glass J.D. (2015). Antecedent Disease is Less Prevalent in Amyotrophic Lateral Sclerosis. Neurodegener. Dis..

[39] Freedman D.M., Kuncl R.W., Cahoon E.K., Rivera D.R., Pfeiffer R.M. (2018). Relationship of statins and other cholesterol-lowering medications and risk of amyotrophic lateral sclerosis in the US elderly. Amyotroph. Lateral. Scler Front. Degener..

[40] Golomb B.A., Verden A., Messner A.K., Koslik H.J., Hoffman K.B. (2018). Amyotrophic Lateral Sclerosis Associated with Statin Use: A Disproportionality Analysis of the FDA’s Adverse Event Reporting System. Drug Saf..

[41] Clemens K.E., Klaschik E. (2008). Morphine in the management of dyspnoea in ALS. A pilot study. Eur. J. Neurol..

[42] Umegaki H., Tagami N. (2008). Anesthetic management of an ALS patient with remifentanil. Masui.

[43] Stephens H.E., Lehman E., Raheja D., Yang C., Walsh S., McArthur D.B., Simmons Z. (2015). Pain in amyotrophic lateral sclerosis: Patient and physician perspectives and practices. Amyotroph. Lateral Scler. Front. Degener..

[44] Mitchell C.S., Cates A., Kim R.B., Hollinger S.K. (2015). Undergraduate Biocuration: Developing Tomorrow’s Researchers While Mining Today’s Data. J. Undergrad. Neurosci. Educ..

[45] Pfohl S.R., Kim R.B., Coan G.S., Mitchell C.S. (2018). Unraveling the Complexity of Amyotrophic Lateral Sclerosis Survival Prediction. Front. Neuroinform..

[46] Strobl C., Boulesteix A.L., Zeileis A., Hothorn T. (2007). Bias in random forest variable importance measures: Illustrations, sources and a solution. BMC Bioinform..

[47] Korner S., Kollewe K., Abdulla S., Zapf A., Dengler R., Petri S. (2015). Interaction of physical function, quality of life and depression in Amyotrophic lateral sclerosis: Characterization of a large patient cohort. BMC Neurol..

[48] Murrell R. (1999). Quality of life and neurological illness: A review of the literature. Neuropsychol. Rev..

[49] Lo Coco G., Lo Coco D., Cicero V., Oliveri A., Lo Verso G., Piccoli F., La Bella V. (2005). Individual and health-related quality of life assessment in amyotrophic lateral sclerosis patients and their caregivers. J. Neurol. Sci..

[50] Gauthier A., Vignola A., Calvo A., Cavallo E., Moglia C., Sellitti L., Mutani R., Chio A. (2007). A longitudinal study on quality of life and depression in ALS patient-caregiver couples. Neurology.

[51] Bourke S.C., Shaw P.J., Gibson G.J. (2001). Respiratory function vs sleep-disordered breathing as predictors of QOL in ALS. Neurology.

[52] Simmons Z. (2005). Management strategies for patients with amyotrophic lateral sclerosis from diagnosis through death. Neurologist.

[53] Burkhardt C., Neuwirth C., Sommacal A., Andersen P.M., Weber M. (2017). Is survival improved by the use of NIV and PEG in amyotrophic lateral sclerosis (ALS)? A post-mortem study of 80 ALS patients. PLoS ONE.

[54] Smith K.L., Cunningham J.I., Eyerman D.J., Dean R.L., Deaver D.R., Sanchez C. (2019). Opioid system modulators buprenorphine and samidorphan alter behavior and extracellular neurotransmitter concentrations in the Wistar Kyoto rat. Neuropharmacology.

[55] Coan G., Mitchell C.S. (2015). An Assessment of Possible Neuropathology and Clinical Relationships in 46 Sporadic Amyotrophic Lateral Sclerosis Patient Autopsies. Neurodegener. Dis..

[56] Kawada T., Thakore N.J., Pioro E.P. (2016). Depression in ALS in a large self-reporting cohort Author Response. Neurology.

[57] Thakore N.J., Pioro E.P. (2016). Depression in ALS in a large self-reporting cohort. Neurology.

[58] Handy C.R., Krudy C., Boulis N., Federici T. (2011). Pain in amyotrophic lateral sclerosis: A neglected aspect of disease. Neurol. Res. Int..

[59] Weishaupt J.H., Bartels C., Polking E., Dietrich J., Rohde G., Poeggeler B., Mertens N., Sperling S., Bohn M., Huther G. (2006). Reduced oxidative damage in ALS by high-dose enteral melatonin treatment. J. Pineal Res..

[60] Bond L., Bernhardt K., Madria P., Sorrentino K., Scelsi H., Mitchell C.S. (2018). A Metadata Analysis of Oxidative Stress Etiology in Preclinical Amyotrophic Lateral Sclerosis: Benefits of Antioxidant Therapy. Front. Neurosci..

[61] De Carvalho M., Swash M. (1998). Fasciculation potentials: A study of amyotrophic lateral sclerosis and other neurogenic disorders. Muscle Nerve.

[62] Shimizu T., Fujimaki Y., Nakatani-Enomoto S., Matsubara S., Watabe K., Rossini P.M., Ugawa Y. (2014). Complex fasciculation potentials and survival in amyotrophic lateral sclerosis. Clin. Neurophysiol..

[63] Gonzalez F. (2009). Diphenhydramine may be useful as a palliative treatment for patients dying with Parkinson’s disease and tremors: A case report and discussion. Am. J. Hosp. Palliat Care.

[64] Kaneb H.M., Sharp P.S., Rahmani-Kondori N., Wells D.J. (2011). Metformin treatment has no beneficial effect in a dose-response survival study in the SOD1(G93A) mouse model of ALS and is harmful in female mice. PLoS ONE.

[65] Scelsa S.N., Khan I. (2000). Blood pressure elevations in riluzole-treated patients with amyotrophic lateral sclerosis. Eur. Neurol..

[66] Shimizu T., Kato S., Hayashi M., Hayashi H., Tanabe H. (1996). Amyotrophic lateral sclerosis with hypertensive attacks: Blood pressure changes in response to drug administration. Clin. Auton Res..

[67] Irvin C.W., Kim R.B., Mitchell C.S. (2015). Seeking homeostasis: Temporal trends in respiration, oxidation, and calcium in SOD1 G93A Amyotrophic Lateral Sclerosis mice. Front. Cell Neurosci..

[68] Herskovits A.Z., Hunter T.A., Maxwell N., Pereira K., Whittaker C.A., Valdez G., Guarente L.P. (2018). SIRT1 deacetylase in aging-induced neuromuscular degeneration and amyotrophic lateral sclerosis. Aging Cell.

[69] Tang B.L. (2017). Could Sirtuin Activities Modify ALS Onset and Progression?. Cell Mol. Neurobiol..

