# Supplementary material for: Associations of Patient Mood, Modulators of Quality of Life, and Pharmaceuticals with Amyotrophic Lateral Sclerosis Survival Duration
Source: Behav Sci (Basel). 2020 Jan 10;10(1):33. doi: 10.3390/bs10010033 (PMC7016647; doi:10.3390/bs10010033)
Supplement: Supplementary file 1 [file behavsci-10-00033-s001.pdf]

behavioral sciences

MDPI

Table S1. Clinical impression of mood (CIM) keyword scoring table. Keyword searches and corresponding clinic impression of mood (CIM) score determinations. Original CIM method as published in Bond, et al., (2019). *Brain Sciences* 9:223.

CIM Score 1 (Negative Mood)	CIM Score 0 (Positive/Neutral Mood)	Expert Investigation of Patient Records to Determine CIM
Anxious	Appropriate	Crying**
Depressed	Cheerful	Decreased*
Dysthymic	Euthymic	Down*
Flat	Fair	Increased*
Frustrated	Good	Laughing**
Irritable	Normal	Stable*
Liable	Pleasant	Tearful**
Not Good		Unstable*
Sad		Up*
Struggling		
Volatile		

* Requires probing of mood at previous appointment. ** Requires probing patient record mention of pseudobulbar affect (PBA), if confirmed, then crying or laughing is not considered as an indicator of mood

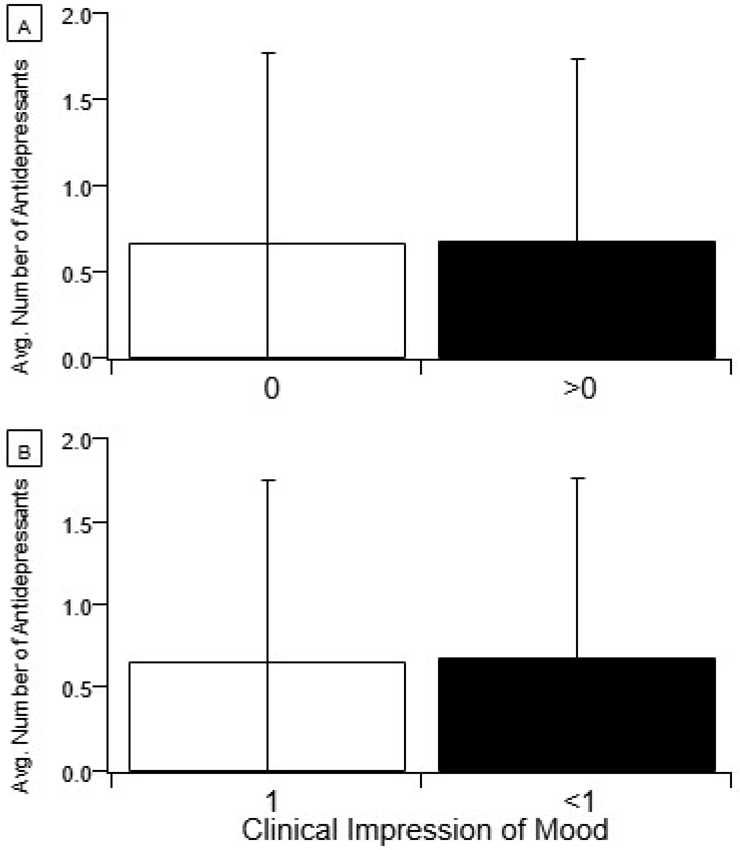

Figure S1. The Clinical Impression of Mood (CIM) does not have an effect on the number of antidepressants taken by ALS patients. A patient's quality of life was quantified using a metric in which the clinicians evaluated patient mood from visit to visit. All antidepressant interventions in a patient's chart were recorded on a per-visit and per-patient basis. ***(A)*** The average number of antidepressants taken by patients with a CIM of zero (n = 569) was not significantly different from the average number taken by those with a CIM greater than zero (n = 577) (p = 0.7877, t-test). ***(B)*** Similarly, the average number taken by patients with a CIM of one (n = 211) was not significantly different than those with a CIM less than one (n = 962) (p = 0.7762, t-test).

behavioral sciences

MDPI

Table S2. Definitions, sample size, and key for the top 8.5% of other pharmaceuticals and supplements. Top 8.5% of medications was determined using a standard statistical power analysis. (Code) referenced corresponds to main article Figure 3.

Intervention Category (Code)	Patients (% of 1585)	Visits (% of 8118)	Definition of Category	Examples of Medications
Rilutek (Riluk)	945 (59.6%)	2874 (35.4%)	Drug used to treat ALS etiology	Rilutek, Riluzole
NSAID (NSAID)	661 (41.7%)	1685 (20.8%)	Analgesia and anti-inflammatory	Aspirin, Ibuprofen, Celebrex, ASA
Stimulant (Stimut)	646 (40.8%)	1703 (21%)	Increase energy or control ADHD	Nuedexta, Adderall
Stimulant laxative (Stimte)	641 (40.4%)	1663 (20.5%)	Induces bowel movements	Senna, Bisacodyl, Dulcolax
Nutrition Supplement (Nutrlt)	618 (39.0%)	1528 (18.8%)	Maintain or gain body weight	IV, Can, Nutrition, Meals
Vitamin (Vitan)	575 (36.3%)	1528 (18.8%)	Individual vitamin supplement	Vitamin
Calcium Supplement (Calcst)	555 (35.0%)	1464 (18%)	Mineral essential to bone, teeth, heart, nerve, blood, thyroid, etc.	Calcium, Caltrate, Citracal, Ca+
SSRI (SSRI)	524 (33.1%)	1494 (18.4%)	Antidepressant, anti-anxiety	Lexapro, Zoloft, Paxil, Citalopram
Opioid (Opiod)	516 (32.6%)	1276 (15.7%)	Strong prescription pain medicine that targets opioid receptors	Lortab, Percocet, Hydrocodone, Oxycodone
Vitamin A (VitaA)	488 (30.8%)	1277 (15.7%)	Supplement promotes retinol or retinoic acid; key to eye health.	Carotene, Betacarotene, Retin, VitA
ACE inhibitor (ACEhr)	469 (29.6%)	1218 (15%)	Vessel dilator to treat hypertension	Lisinopril, Lotrel, Benazepril, Enalapril
Benzodiazepine (Benzae)	440 (27.8%)	1203 (14.8%)	Reduces overall neural activity	Clonazepam, Klonopin, Lorazepam
Potassium-sparing diuretic (Potasd)	422 (26.6%)	1015 (12.5%)	Promotes diuresis and homeostasis	Hydrochlorothiazide, HCTZ, Spironolactone, Triamterene
Beta blocker (Beta)	394 (24. 9%)	1004 (12.4%)	Manage cardiac arrhythmias	Metoprolol, Atenolol, Toprol
Omega 3 (Omeg3)	371 (23.4%)	986 (12.1%)	Polyunsaturated fat supplement for cardiovascular and brain health	Fish, Fatty, Oil, Omega
Multivitamin (multvn)	359 (22.6%)	888 (10.9%)	Prophylactic dietary supplement	Multivitamin, Centrum Silver
Statin (Statn)	346 (21.8%)	844 (10.4%)	Used to treat hyperlipidemia.	Simvastatin, Lipitor, Atorvastatin,
Antispastic (Antipc)	340 (21.4%)	1150 (14.2%)	Suppresses muscle spasms.	Baclofen, Baclofen, Soma
General sleep (Gene)	322 (20.3%)	767 (9.4%)	Any agent that reduces insomnia	Zolpidem, Provigil, Lunesta
Irrev. COX inhibitor (ici)	319 (20.1%)	774 (9.5%)	Analgesic or anti-clot agent	Aspirin, Bayer, ASA81, Asprin
Antihistamine (antihe)	313 (19.7%)	735 (9.1%)	Treats seasonal allergies	Claritin, Diphenhydramine, Promethazine, Allegra
Proton pump inhibitor (ppi)	290 (18.2%)	700 (8.6%)	Reduces gastric acid production	Omeprazole, Nexium, Prilosec

Calcium channel blocker (ccb)	287 (18.1%)	737 (9.1%)	Primarily treats hypertension.	Norvasc, Diltiazem, Verapamil
Acetaminophen (acerin)	282 (17.8%)	631 (7.8%)	Analgesic and anti-pyrexia agent	Lortab, Acetaminophen, Vicodin,
Feeding tube (feedge)	266 (16.8%)	517 (6.4%)	Tube to stomach to supply nutrition	Enteral, Tube, Vivelle, NG
Alternative Herbal Medication (Ahmn)	246 (15.5%)	510 (6.3%)	Therapuetic use of plants or herbs	Grape, Garlic, Herbal, Ginkgo

behavioral sciences

MDPI

Table S3. Prevalent pharmaceuticals and supplements, which did *not* have a significant association with survival duration.

Category	F ratio	M ratio	sex p-val	bulbar ratio	limb ratio	onset p-val	mean user age (yrs)	age p-val	short dur ratio	long dur ratio	surv dur p-val	△ surv (mo.)
corticosteroid	0.12	0.13		0.11	0.13		59.5		0.11	0.15		7.0
steroid	0.14	0.14		0.12	0.14		59.3		0.12	0.17		6.2
omega 3	0.26	0.22	*	0.20	0.25		59.2		0.18	0.23		6.5
antacid	0.09	0.08		0.09	0.09		62.9	*	0.07	0.11		5.7
benzodiazepine	0.30	0.26	*	0.24	0.30	*	57.9	**	0.25	0.30		6.7
Rilutek	0.58	0.61	*	0.59	0.61		58.9	**	0.58	0.63		1.3
acetaminophen	0.20	0.17		0.16	0.19		57.9	**	0.14	0.17		5.4
potassium sparing diuretic	0.13	0.14		0.13	0.14		64.1	**	0.11	0.15		0.0
proton pump inhibitor	0.20	0.17	*	0.21	0.17		63.5	**	0.16	0.20		3.6
opioid	0.37	0.30	**	0.29	0.35	*	58.5	**	0.28	0.33		6.6
expectorant	0.13	0.11		0.15	0.10	*	60.1		0.09	0.12		1.5
calcium supplement	0.38	0.33	*	0.33	0.36		60.7		0.29	0.33		3.5
ace inhibitor	0.29	0.30		0.29	0.31		61.1		0.25	0.29		2.7
general hypothyroidism	0.16	0.05	**	0.12	0.08	*	65.2	**	0.08	0.10		9.1
diuretic	0.29	0.25	*	0.29	0.26		64.0	**	0.25	0.28		1.0
thiazide diuretic	0.16	0.11	*	0.16	0.13		63.9	**	0.14	0.11		−2.4
potassium-sparing diuretic	0.17	0.11	**	0.16	0.13		64.0	**	0.13	0.11		−5.2
nsaid	0.44	0.40	*	0.40	0.43		61.6	**	0.41	0.43		3.0
quinolone antibiotic	0.09	0.08		0.08	0.10		61.0		0.08	0.09		5.1
statin	0.21	0.23		0.26	0.21		65.1	**	0.23	0.21		−3.7
ssri	0.39	0.29	**	0.34	0.34		60.1		0.35	0.37		2.4
calcium channel blocker	0.19	0.17		0.20	0.18		60.8		0.15	0.17		−0.8
general urinary tract	0.08	0.14	**	0.09	0.12		64.9	**	0.11	0.10		−1.5
levothyroxine	0.16	0.06	**	0.12	0.08	*	65.1	**	0.09	0.09		4.9
irreversible cyclooxygenase inhibitor	0.17	0.22		0.21	0.20		64.6	**	0.21	0.20		2.1
decongestant	0.13	0.09	*	0.12	0.10		60.4		0.09	0.08		−1.1
vitamin b	0.13	0.11		0.10	0.13		61.8	*	0.09	0.09		0.8
beta blocker	0.27	0.24	*	0.24	0.26		63.1	**	0.25	0.25		1.4
potassium	0.28	0.25	*	0.28	0.26		63.9	**	0.25	0.25		−2.0

F is for female, M for male, "short dur" is for short survival duration, "long dur" is for long survival duration, "△ surv" is change in survival duration (in months), * denotes low significance threshold of 0.05 > p-value ≥ 0.001 and ** denotes high significance threshold where p-value < 0.001. Ratio is ratio of users to non-users with a specific characteristic.
